# Out-of-pocket costs for patients with psoriasis in an outpatient dermatology referral service^☆^^☆☆^

**DOI:** 10.1016/j.abd.2020.09.004

**Published:** 2021-03-07

**Authors:** Ana María Maya-Rico, Ángela Londoño-García, Arlex Uriel Palacios-Barahona, Sol Beatriz Jimenez-Tamayo, Estefanía Muriel-Lopera

**Affiliations:** Universidad CES, Medellín, Colombia

**Keywords:** Health care costs, Health expenditures, Psoriasis

## Abstract

**Background:**

Psoriasis is a chronic disease that derives great costs to the health care system. In Colombia, due to deficiencies in this system, patients are more likely to incur in out-of-pocket expenses; money that has never been quantified in this country.

**Objectives:**

To quantify out-of-pocket expenses and to analyze their relation to patients' clinical and labor characteristics in a cohort of psoriatic patients.

**Methods:**

A single-center, cross-sectional study was performed, evaluating psoriasis patients.

**Results:**

A total of 100 psoriasis patients were analyzed. We identified that patients with higher dermatology life quality index and in phototherapy treatment were the ones that had higher out-of-pocket costs (p = 0.006 and 0.005, respectively). We found no correlation between out-of-pocket costs and occupational status, psoriasis area severity index or other types of treatment. The largest amount of money was used to buy medications and bus transportation with a maximum up to 440.50 and 528.60 USD, respectively. Among the 100 participants the total expense was 11131.90 USD in a 6-month period.

**Study limitations:**

Lack of measurement of the labor productivity and labor absenteeism secondary to sick leave.

**Conclusion:**

Out-of-pocket costs are similar with what was shown in previous studies. We found statistically significant differences for the DLQI in comparison with out-of-pocket expenses, regardless of the PASI level. Phototherapy treatment also had statistically significant differences in relationship with out-of-pocket expenses, when compared to other treatments, because it requires higher expenses in transportation, copayments, and alimentation during appointment assistance.

## Introduction

Psoriasis is a chronic disease that affects primarily the skin and joints, with an estimated global prevalence between 0.6% and 6.5%.^1^ In Colombia, psoriasis has an estimated burden of 43.6 DALY (Disability-Adjusted Life-Years) per 100,000 people, and about 3% of consultations to Dermatology services.^2^, ^3^ Among patients with moderate to severe psoriasis, the disability is comparable to other chronic conditions such as rheumatoid arthritis, diabetes mellitus and multiple sclerosis; and its treatment is sometimes challenging.^3^ Due to its multisystemic compromise, several considerations must be accounted for its treatment, not only cutaneous and articular, and this undeniably translates into costs for both the health care system and patients: multiple or combined treatments, more expensive treatments, outpatient and emergency services consultations due to the disease or its complications, and hospitalizations that could be prolonged.^3^ In the United States, the yearly total of all-cause health care cost was between 33.82 USD (United States Dollars) for discontinuers and 60.73 USD for switchers, with around 20% not related to prescription drug costs.^4^

The economic aspects of health are increasingly important in all health care systems.^5^ In Colombia, due to deficiencies in the health care system, patients are more likely to incur in out-of-pocket expenses.^6^ Health related out-of-pocket expenses refers to expenses related to transportation to medical appointments, food, co-payments, the purchase of medications or any service or exam that is not covered by the health care system and must be paid by the patients themselves.^7^ Particularly, incurring in these expenses seem to be catastrophic due to the lack of health coverage in unemployed patients.^6^ So far, cost studies in psoriasis have been performed mainly alongside the evaluation of effectiveness of biological treatments. Cost studies in daily clinical practice are scarce, and the few existing ones focus on the direct costs of the disease with little but important information about indirect costs.^5^ For instance, annual indirect costs attributable to psoriasis range between 23.9 and 35.4 billion dollars in the United States, which reiterates the substantial economic burden secondary to this disease.^8^

As there is a lack of Colombian evidence related to the behavior of out-of-pocket expenses among psoriasis patients, the aim of this study was to quantify these costs and to analyze their relation to patients’ clinical labor characteristics in a cohort of psoriatic patients from a dermatological outpatient clinic.

## Methods

Single-center, cross-sectional study carried out at the CES University School of Medicine’s Dermatological Center “CES Sabaneta” (Sabaneta, Colombia) from September 2018 to March 2019.

All patients older than 18 years with a diagnosis of mil and moderate-severe psoriasis in any of its clinical variants were considered eligible and invited to participate. Patients were excluded if they refused to participate or if they could not attend study appointments. Sample size at convenience. All patients with psoriasis were identified and invited to participate in the study.

The primary outcome was to measure out-of-pocket expenses in patients with psoriasis. Out-of-pocket costs were measured by a survey (Appendix 1) that took into account quantitative economic variables such as: total money spent in the last 6-months on medical products such as thermometers, syringes, needles, bandages, gauze, cotton, medications, medical appointments, food bought during the attendance to medical appointments, co-payments for medical or phototherapy appointments, private medical appointments for general medicine, specialists, alternative medicine and/or psychology, transportation (bus, taxi, subway, personal vehicle, subway, walking, or other), and/or lodging, in case of appointments in other cities or municipalities. The medical products were calculated according to pharmacy prices and the money was measured in Colombian Pesos (COP) and converted to US Dollars (USD) according to the representative exchange rate of the market (Source: Banco de la República de Colombia exchange value: 3177.94 COP April 24, 2019).

Other economic and clinical variables like employment status (employee, unemployed or pensioner), cause of unemployment if applied (due to illness or other causes), work absenteeism measured in number of days that the patient did not go to work due to the disease in the last 6-months with or without sick leave; Area and Severity Index of Psoriasis (PASI), Dermatological Quality of Life Index (DLQI) which evaluates 10 questions in relation to the impact of cutaneous disease on the social, family, sexual and labor spheres, responding: very much (3 points), a lot (2 points), a little (1 point) and not at all (0 points), giving a final score between 0–30 with 0 being the least affected in life quality and 30 the maximum; the course time of the disease in years, comorbidities (diabetes, hypertension, dyslipidemia, metabolic syndrome, cardiovascular disease, obesity, cancer, others), type of treatment (topical, systemic, phototherapy, biological, others or none), and cause of treatment abandonment if applied (costs, transportation, poor attention, lack of availability of appointments, disaffiliation of health service provider, other causes) were also considered in the survey.^9^, ^10^

All patients with psoriasis were invited to participate and underwent a complete physical examination and a survey, which were performed by a trained third-year dermatology resident. To reduce information biases, patients were sensitized about the relevance of information veracity. A pilot test was conducted in 5 patients in order to determine the examiner’s performance under standardized protocol and to verify the patients’ understanding of the study aim and required information.

Descriptive analysis of out-of-pocket expenses, sociodemographic and clinical patient characteristics was performed using absolute and relative frequencies for the qualitative variables, and mean, standard deviation median and range for the quantitative ones. The relation of patient characteristics with out-of-pocket expenses was analyzed with generalized linear models, family gaussian, link identity; crude and adjusted mean differences by PASI, DLQI, occupational status, treatment with 95% Confidence Intervals (95% CI) and p-values are presented. Statistical significance was set to 0.05. Statistical analyses were performed in SPSS version 21 licensed by the CES University. This study was approved by the research and ethics committee of the CES University.

## Results

Out of 100 patients who participated in the study, 49% were women. The average age was 54.7 (SD = 13.1) years (Table 1). The level of education showed in equal proportions patients with higher education (college) versus patients with elementary, middle, high school and without any education; 99% of patients were affiliated to a health care system, 80% of them as contributors. 54% of the patients were employed, of whom 8 missed work, 3 with sick leave and 5 without it. The remaining 46% were pensioners or unemployed in the same proportion, only 2% attributing it to psoriasis. The mean PASI and DLQI was 4.8 and 7.2, respectively, as shown in Table 1.

**Table 1:** Sociodemographic and clinical characteristics of the participants (n = 100).

	n	%
**Sex**		
Female	49	49
Male	51.0	51.0
Age^a^	54.7 (13.1)	
**Educational level**		
None	1.0	1.0
Less than high school	18.0	18.0
High school graduate	31.0	31.0
Some University	23.0	23.0
University and Graduate school	27.0	27.0
**Health care system affiliation**		
Contibutor	80	80
Beneficiary	19	19
None	1	1
Occupational status		
Employee	54	54
Unemployed	23	23
Pensioner	23	23
**Cause of unemployment or pension**		
Disease (psoriasis)	2	2
Other	44	44
Employee	54	54
**Work absenteeism**		
Never missed work	46	46
Missed work with “sick leave”	3	3
Missed word without “sick leave”	5	5
Unemployed or pensioner	46	46
PASI^a^	4.8 (5.8)	
DLQI^a^	7.2 (7.0)	
Time since diagnosis (years)^a^	15.3 (12.8)	
**Comorbidities**		
None	46	46
Diabetes *mellitus*	14	14
Arterial hypertension	20	20
Dyslipidemia	24	24
Metabolic syndrome	6	6
Cardiovascular disease	0	0
Obesity	12	12
Cancer	1	1
Other	19	19
**Treatment**		
Topical	87	87
Systemic	20	20
Phototherapy	13	13
Biological	14	14
Other	7	7
None	7	7

a Median (interquartile range).

The largest amount of out-of-pocket money was used to purchase medications and bus transportation with a maximum of up to 440.50 and 528.60 USD each, respectively (Table 2). In bus transportation, patients spent an average of 13.38 USD. The mean out-of-pocket expense related to medical care measured in 2019 USD for medical products, medications, exams, and alimentation was 1.85, 63.20, 1.50, and 2.35 USD, respectively. For medical co-payments for appointments attendance or phototherapy was 3.36 and 0.57 USD. Particular appointments of family medicine, specialists, alternative medicine and psychology had a mean of 2.77, 2.02, 1.82 and 0.62 USD respectively. Among the 100 participants, the total expense was of 11,131.90 USD in the 6-month period.

**Table 2:** Out-of-Pocket expenses (US dollars, 6-month period) related to medical care.

	Mean	Standard deviation	Median	Minimum	Maximum
**Medical products**	1.85	7.27	0	0	61.10
**Medication**	63.20	82.70	36.60	0	440.50
**Exams**	1.50	3.90	0	0	25.20
**Alimentation**	2.35	6.41	0	0	45.30
**Medical appointments copays**	3.36	8.33	0.90	0	62.90
**Phototherapy appointments copays**	0.57	2.94	0	0	22.00
**Private practice appointments**					
Family medicine	2.57	16.97	0	0	141.60
Specialists	2.02	8.40	0	0	44.10
Alternative medicine	1.82	8.78	0	0	62.90
Psychology	0.62	6.29	0	0	62.90
**Transportation**					
Bus	13.38	59.26	0	0	528.60
Taxi	5.30	27.06	0	0	254.90
Metro	8.27	31.16	0	0	226.60
Own vehicle	4.40	24.26	0	0	182.80
Lodging	0	0	0	0	0

The differences in means in factors associated with out-of-pocket expenses related to medical care are shown in Table 3. First, a crude analysis of the variables was performed, with the PASI and phototherapy variables being significant. When adjusting the model for the PASI, DLQI, occupational status and treatment variables, only the DLQI and phototherapy variables were significant. For the DLQI, the mean difference was 5.42 with a 95% Confidence Interval (95% CI: 1.57–9.28); this means that for every one-point increase in the DLQI, the out-of-pocket spending increases by 5.42 USD over a 6-month period. Additionally, phototherapy also had a statistically significant difference in means, compared to not having any type of treatment, with a mean difference of 33.38 (95% CI: 8.64–58.14).

**Table 3:** Factors associated with out-of-pocket expenses (US Dollars, 6-month period) related to medical care^a^.

Variables in the model	Observed(crude)			Adjusted^b^		
	Mean difference	95% CI	p-value	Mean difference	95% CI	p-value
PASI	6.25	(1.92–10.57)	0.005	2.14	(−2.62–6.91)	0.374
DLQI	6.95	(3.53–10.37)	0	5.42	(1.57–9.28)	0.006
Occupational status						
Employee	0			0		
Unemployed	−31.84	(−95.45–31.75)	0.323	−20.17	(−79.18–38.84)	0.499
Pensioner	−55.55	(−119.15–8.053)	0.086	−44.97	(−79.18–38.84)	0.133
Treatment						
Topical	61.13	(−50.81–173.07)	0.281	33.37	(74.08–140.84)	0.539
Systemic	−7.86	(−39.42–23.68)	0.622	−14.53	(−44.59–15.52)	0.339
Phototherapy	33.38	(8.64–58.14)	0.009	33.88	(10.65–57.12)	0.005
Biological	−3.38	(−22.85–16.09)	0.731	−2.81	(−21.57–15.94)	0.766
Other	−7.75	(−32.29–16.78)	0.532	−4.61	(−70.98–159.10)	0.694
None	0			0		

95% CI, Confidence Interval 95%.

a Data using a generalized linear models’ family gaussian, link identity.

b Adjusted by: PASI, DLQI, occupational status, treatment.

## Discussion

In this study, we aimed to quantify the out-of-pocket costs of patients with psoriasis and analyze their relationship with the clinical characteristics of the patients' work in a cohort of a dermatological outpatient clinic. We identified that the main out-of-pocket expense is in medication, transportation and copays for medical appointments, and the main characteristics associated with out-of-pocket expenses were DLQI and phototherapy treatment. We documented a total of 11,131.90 USD spent in the last 6-months among the 100 participants. The largest amount of out-of-pocket money was used to purchase medications and transportation with a maximum of 440.50 and 528.60 USD, respectively. In bus transportation, patients spent an average of 13.38 USD. The average spent money was 111.30 USD per patient in the 6-month period. Our findings are consistent with previous studies where a similar behavior in out-of-pocket expenditures has been documented. In a study conducted in the United States, Javitz et al. reported similar out-of-pocket expenses of 356.00 USD per patient per year.^11^

Other studies have shown out-of-pocket expenses such as 706.00 USD per patient in a 6-month period,^3^ and even 1,255.00 USD vs. 913.00 USD in non-psoriatic patients with a stadistically significant p of 0.001.^12^ Also, Gunnarson described an Australian study that reported annual out-of-pocket expenses for Over The Counter (OTC) and prescription medications of approximately 250.00 AUD (Australian Dollar) per person during 1997–1999.^12^ In the United States, studies have also been conducted measuring both direct and indirect costs with regard to psoriasis, the largest so far published in 2008, where it was found that indirect costs were up to 2,748.00 USD per year per worker, even overcoming serious gastrointestinal diseases such as ulcerative colitis, gastroesophageal reflux, diverticular disease and peptic ulcer disease; and being comparable with those described in the Schmitt and Ford study, in which they report 2571.00 USD per worker per year.^13^, ^14^

In Switzerland, the cost of out-of-pocket expenses and outpatient visits in the year 2005 amounted from 1,141.00 to 7,957.00 Euros per patient per year.^15^ Moreno et al. considered indirect costs related to sick leave, early retirement, patient assistance and out-of-pocket expenses paid by the patient due to Psoriatic Arthritis (PsA) and presented an average annual indirect cost per patient of 2,066.29 USD.^16^ Kawalec et al. also documented higher out-of-pocket expenses paid by the patient due to PsA, with mean annual indirect cost per patient to reach 1,261.00 Euros.^17^ The present study found lower expenses because the authors did not measure indirect costs in the form of work absenteeism and did not have any patients with PsA.

The present study showed a mean PASI and DLQI of 4.8 and 7.2, respectively. In a study conducted in Colombia in 2014 about direct costs of psoriasis, the mean PASI and DLQI documented were 5.7 and 8.2, respectively.^5^ In our case, a DLQI of 7.2 on average expresses moderate impact of life quality, however it did not correlate with PASI level, which can be explained because a significant number of our participants had only palmoplantar involvement. This reassures the great impairment in life quality, regardless of the PASI level or severity of the disease. That component alone can have an impact on the labor sphere of the patients, as Mansouri et al. showed in their study about work and sickness absence among psoriasis patients, where the majority of patients (68%) had PASI levels under 10.^18^, ^19^

We also found statistically significant differences for the DLQI in relation to the out-of-pocket expenses, and this can be explained because the greater the impact on the quality of life, the sicker the patients feel, and they consequently buy more medication or have greater attendance at appointments, regardless of the PASI level. This outcome could also be explained in an inverse manner: with higher expenses, greater impact on life quality. Reviewing the literature, we did not find studies comparing out-of-pocket expenses with quality of life; however, in the few that we find regarding indirect costs and impact On Life Quality (QoL), it is well known that these financial burdens are associated with lower quality of life in patients, and that the psychological impact of psoriasis may play a role in patients' work productivity and therefore in their income.^14^, ^20^, ^21^

Schöffski et al., found that the patients with higher DLQI were the ones who had higher costs, however in this study, unlike us, direct costs were also included.^22^ Additionally, in a study by Hawro et al. 23% of patients report that psoriasis has negatively influenced their choice of a professional career; they propose that psoriasis results in stigmatization with social rejection and psychological distress, as well as with subsequent long-term behavioral changes that lead to a lower economic status and a negative impact on patients’ QoL, meaning they can even spend more time in therapy, which can be translated, in our case, into higher expenses in OTC medication.^23^

A systematic review suggested that the more severe the disease, the more out-of-pocket expenses are had; additionally, being a disease associated with multiple comorbidities, this increases indirect costs considerably.^8^, ^24^ This does not correlate with our study, since we do not find statistically significant differences between the out-of-pocket expenses and the different PASI levels, which may be different from other studies but not comparable, since they take into account work productivity rather than out-of-pocket expenses; like Ayala et al. that report that lower QoL at work and financial status/income were correlated with severity of psoriasis.^25^

In our study, 93% of the patients were in treatment, 34% with oral or biological treatment and most of the patients (87%) were also receiving topical treatment. This agrees with world literature in which topical is the main treatment.^3^, ^20^ Regarding treatment and costs, phototherapy had a statistically significant difference in means, compared to not having any type of treatment. We propose that it increases the out-of-pocket expenses explained by the attendance to phototherapy appointments, which are on average 2–3 times a week, which translates into higher expenses in transportation, copayments, and alimentation during appointment assistance. Other studies have also found a relation in terms of indirect costs increasing significantly only in the case of phototherapy, when compared to other treatment modalities.^26^

As a limitation in our study, we identify the lack of measurement regarding the loss of labor productivity and labor absenteeism translated into money lost in sick leave. Nevertheless, to our knowledge, this is the first study of out-of-pocket costs in psoriasis in Colombia, a country where people allocate 3.1% of their total expenses to health payments, a considerable percentage^7^ Out-of-pocket costs are important for patients because in large measure, they are unaware of the costs for different psoriasis treatments, and besides efficacy and safety, out-of-pocket costs are one of the most important factors when deciding on a psoriasis treatment.^27^

Recent studies about indirect costs in patients with psoriasis described by Villacorta, Feldman and Lopes focus on work productivity loss, whereas our study provides additional information about out-of-pocket costs assumed by the patient such as medications and transportation to phototherapy sessions.^28^, ^29^, ^30^

## Conclusion

The out-of-pocket costs are similar to with pasts studies. We found statistically significant differences for the DLQI in relation with the out-of-pocket expenses, regardless of the PASI level. Phototherapy treatment also had a statistically significant difference in relation with the out-of-pocket expenses when compared to other treatments, because it requires higher expenses in transportation, copayments, and alimentation during appointment assistance. We found no correlation between out-of-pocket costs and occupational status, PASI or other types of treatment. The largest amount of money was used to buy medications and bus transportation. Among the 100 participants, the total expense was of 11,131.90 USD during the 6-month period.

## Financial support

CES University, Medellín, Colombia.

## Authors’ contributions

Ana María Maya-Rico: Design and planning of the study; drafting and editing of the manuscript; collection, analysis, and interpretation of data; critical review of the literature.

Ángela Londoño-García: approval of the final version of the manuscript; design and planning of the study; drafting and editing of the manuscript; effective participation in research orientation; critical review of the manuscript.

Arlex Uriel Palacios-Barahona: Statistical analysis; approval of the final version of the manuscript; design and planning of the study; effective participation in research orientation; critical review of the literature; critical review of the manuscript.

Sol Beatriz Jimenez-Tamayo: Approval of the final version of the manuscript; design and planning of the study; effective participation in research orientation; critical review of the manuscript.

Estefanía Muriel-Lopera: Drafting and editing of the manuscript; collection, analysis, and interpretation of data; critical review of the literature.

## Conflicts of interest

None declared.
